# Effectiveness of Intermittent Hypoxia–Hyperoxia Therapy in Different Pathologies with Possible Metabolic Implications

**DOI:** 10.3390/metabo13020181

**Published:** 2023-01-25

**Authors:** Andreea-Bianca Uzun, Mădălina Gabriela Iliescu, Liliana-Elena Stanciu, Elena-Valentina Ionescu, Rodica Ana Ungur, Viorela Mihaela Ciortea, Laszlo Irsay, Irina Motoașcă, Marius Nicolae Popescu, Florina Ligia Popa, Loredana Pazara, Doina-Ecaterina Tofolean

**Affiliations:** 1 Department of Pulmonology, Faculty of Medicine, Ovidius University of Constanta, 1 University Alley, Campus—Corp B, 900470 Constanta, Romania; 2 County Clinical Emergency Hospital of Constanta, 145 Tomis Blvd., 900591 Constanta, Romania; 3 Department of Physical Medicine and Rehabilitation, Faculty of Medicine, Ovidius University of Constanta, 1 University Alley, Campus—Corp B, 900470 Constanta, Romania; 4 Department of Rehabilitation Medicine, University of Medicine and Pharmacy “Iuliu Hatieganu”, 8 Victor Babes Street, 400012 Cluj-Napoca, Romania; 5 Clinical Rehabilitation Hospital, 46-50 Viilor Street, 400066 Cluj-Napoca, Romania; 6 Department of Rehabilitation Medicine, “Carol Davila” University of Medicine and Pharmacy, 37 Dionisie Lupu Street, 020021 Bucharest, Romania; 7 Physical Medicine and Rehabilitation Department, Faculty of Medicine, “Lucian Blaga” University of Sibiu, Victoriei Blvd., 550024 Sibiu, Romania; 8 Department of Physiopathology, Faculty of Medicine, Ovidius University of Constanta, 1 University Alley, Campus—Corp B, 900470 Constanta, Romania

**Keywords:** intermittent hypoxia hyperoxia, training, physical exercise, rehabilitation

## Abstract

Intermittent oxygen therapy (IHT), initially used in the hypoxic administration variant, has been shown to be effective in various pathologies studied, from cardiopulmonary to vascular and metabolic pathologies and more. IHT used to prevent and treat various diseases has thus gained more and more attention as the years have passed. The mechanisms underlying the beneficial effects have been investigated at multiple biological levels, from systemic physiological reactions to genomic regulation. In the last decade, a new method of intermittent oxygen therapy has been developed that combines hypoxic and hyperoxic periods. They can be applied both at rest and during physical exercise, hence the specific indications in sports medicine. It has been hypothesized that replacing normoxia with moderate hyperoxia may increase the adaptive response to the intermittent hypoxic stimulus by upregulating reactive oxygen species and hypoxia-inducible genes. This systematic literature review is based on the “Preferred Reporting Items for Systematic Reviews and Meta-Analysis”—“PRISMA”—methodology, the widely internationally accepted method.

## 1. Introduction

Intermittent hypoxia (IH) is generally defined as repeated episodes of hypoxia interspersed with normoxic episodes. Experimentally repeated short-term hypoxia (about 5 min) with normoxic intervals has been used by Russian doctors for many years [1]. The first steps in the use of intermittent hypoxia training (IHT) were for the training of athletes, climbers and pilots. Research conducted in 1939–1943 showed that even a small height improves pulmonary ventilation and increases hemoglobin concentration and oxygen saturation. Research at the time also drew attention to the possible curative effects of hypoxic adaptation [2]. Thus, intermittent hypoxia training (IHT) was initially recognized in sports medicine as a potentially useful strategy for improving exercise performance in athletes. In addition to improving physical performance and preclinical protective effects of IHT, it has been considered in healthy subjects to be potentially useful for improving physiological functions as well [3]. IHT is the emerging therapeutic modality for the prevention and treatment of various diseases, gaining increasing attention in recent years [4].

Increased exercise tolerance in patients with cardiovascular, bronchopulmonary and metabolic syndromes improved cardiometabolic status in elderly patients and increased cognitive potential in Alzheimer’s disease and in clinical observations; all of these achieved with the help of IHT have been demonstrated by several studies throughout the years [5].

Medical protocols for intermittent hypoxia therapy have been developed, with this therapy advancing rapidly in modern times. They use mild, non-harmful hypoxic training that provides benefits and drug-free treatment for several chronic degenerative pathologies [6].

In the last decade, intermittent hypoxia–hyperoxia training (IHHT) has entered medical practice, and periods of breathing atmospheric air have been replaced by breathing a hyperoxic gas mixture [7]. A new training method has been suggested, IHHT (intermittent hypoxia–hyperoxia training), which uses hyperoxic intervals instead of normoxic ones between hypoxic breathing sessions. The patient receives a gas mixture containing 30–40% O_2_ in the mask. The efficacy, safety, and tolerability of IHHT have been demonstrated in placebo-controlled pilot clinical trials [8].

Periods of intermittent hypoxia–hyperoxia can be applied as a passive intervention with patients at rest (IHHE—intermittent hypoxic–hyperoxic exposure) or during exercise (IHHT—intermittent hypoxic–hyperoxic training). Intermittent hypoxia–hyperoxia, either passive or in combination with exercise, appears to be a promising therapeutic strategy for various populations [9].

## 2. Experimental Design

This systematic literature review is based on the “Preferred Reporting Items for Systematic Reviews and Meta-Analysis”—“PRISMA”—methodology, the widely internationally accepted method. For the selection by the PRISMA method, in our research, we considered articles indexed in the following international databases: National Center for Biotechnology Information (NCBI)/PubMed, Elsevier, Cochrane and Physiotherapy Evidence Database (PEDro). In addition, articles were selected via a direct internet search (Google search).

Our research went through stages described in the PRISMA flow diagram using the following keywords: intermittent hypoxia–hyperoxia, intermittent hypoxia–hyperoxia training and intermittent hypoxic training (Table 1).

## 3. Results

**Figure 1:**
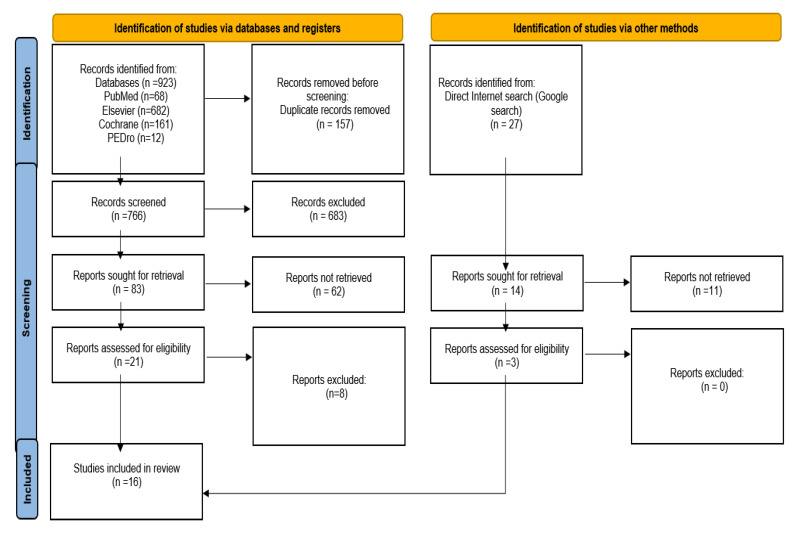
Our adapted PRISMA type of the flow diagram.

We selected articles written in English, published in the mentioned databases, and we identified 923 articles. Appling the PRISMA selection filters resulted in 13 published qualified studies. We added another three free papers found based on a Google search by a direct internet search, which were highly related to our research (Figure 1). 

This article reviews clinical studies on the effect of intermittent hypoxia–hyperoxia and hypoxia–normoxia on various pathologies. Sixteen eligible studies were identified (as shown in Table 2 and Table 3).

Evidence-based literature data focused on the benefits of intermittent hypoxia–hyperoxia therapy (Table 2).

The review outlines the current evidence-based clinical benefits of intermittent hypoxia–hyperoxia in various pathologies by analyzing research reported between 2017 and 2022 in the online database.

In a non-randomized controlled study conducted in 2017, O. Glazachev et al. hypothesized that intermittent hypoxia–hyperoxia training (IHHT) improves exercise tolerance, quality of life and the cardiometabolic profile in patients with coronary artery disease. The study included a total of 46 participants who were assigned to two groups: 27 patients were included in the IHHT group and 19 patients were included in the sham IHHT group. Patients in the IHHT group were to begin a cardiac rehabilitation program, while those in the sham IHHT group completed an 8-week twice-weekly cardiac rehabilitation program. The study was blinded. The schedule that the patients in the IHHT group undertook was as follows: customized repeated exposures to hypoxia (10–12% O_2_) and hyperoxia (30–35% O_2_), three sessions per week, five to seven periods of hypoxia that lasted 4 to 6 min, with 3 min hyperoxic recovery intervals. They attended 15 sessions in total. The schedule of patients in the sham group was similar to that of the IHHT group, but they were exposed to normoxic air (21% O_2_). The conclusions of the study are that an IHHT program is safe and effective for cardiac patients, and it can also improve exercise capacity, lipid profile and quality of life for these patients [10].

Another randomized controlled double-blind trial conducted in 2017 by Bayer et al. hypothesized that IHHT + MTI (multimodal training intervention) would improve exercise tolerance and cognitive function in geriatric patients more than MTI alone. The 34 patients participating in the study were randomly assigned to the hypoxic or normoxic group. The age of the participants was between 64–92 years. All patients participating in the study followed the same MTI for 15–20 days depending on the needs of each, 2–3 days per week, over a period of 5–7 weeks. The MTI consisted of physiotherapy, occupational therapy and cycling. IHHT was performed simultaneously with MTI. Patients in the hypoxic group received hypoxic gas mixtures (10–14% O_2_) for 4–7 min, followed by hyperoxic gas mixtures (30–40% O_2_) for 2 to 4 min. The duration of each session was 30–40 min, including four–eight cycles of hypoxia–hyperoxia. Patients in the normoxic group breathed a normoxic gas mixture (placebo). In total, 12–15 hypoxic or normoxic treatment procedures were performed for both groups, always performed with MTI on the same day. As a result of the study, it was found that IHHT was well tolerated by geriatric patients and could significantly contribute to the improvement of cognitive performance, as well as functional exercise capacity in geriatric patients also subjected to an MTI [11].

In 2018, Dudnik et al. published a study that aimed to investigate the effects of intermittent hypoxia–hyperoxia on CRF (cardiorespiratory fitness) in elderly patients with comorbidities compared to a traditional exercise-based fitness program. A total of 29 cardiac patients with various comorbidities such as type II diabetes, hypertension, chronic obstructive pulmonary disease (COPD), dyslipidemia and mild obesity were randomly assigned to either the IHHT group or the control group. Patients in the IHHT group completed a 5-week program while resting: hypoxia (11–12% O_2_) and hyperoxia (30–33% O_2_). Training consisted of three sessions per week, including 5–7 periods of hypoxia of 4–6 min, then 3 min of hyperoxia. They performed 15 sessions in total. Patients in the control group completed a customized cardiopulmonary exercise program for 8 weeks. Participants were exposed to 15 sessions of simulated hypoxia for 8 weeks (breathing room air), twice a week. The authors concluded that IHHT could be an option for the elderly who are unable to exercise, being a safe alternative in these patients. IHHT for 5 weeks is as effective as an 8-week exercise program in improving CRF, without hematological changes [12].

Tuter et al., also in 2018, performed a prospective, randomized controlled trial on patients with ischemic heart disease and indication for coronary artery bypass graft (CABG) surgery. The primary endpoint of this study was the serum concentration of troponin I and lactate at 2 and 24 h after surgery. Patients were randomly assigned to one of three groups. Patients in the IHHT group underwent four daily hypoxia–hyperoxia procedures before CABG surgery. They received hypoxia (12% O_2_) for 3–5 min and hyperoxia (35–40% O_2_) for 1–3 min. Each procedure lasted between 20–30 min. Patients in the RIP (remote ischemic preconditioning) group underwent RIP before anesthesia and skin incision. Patients in the IHHT control group also underwent four daily procedures before surgery, with the training period being 40 min, simulating IHHT. This study demonstrated the safety and efficacy of intermittent hypoxia–hyperoxia as a method of preconditioning and cardioprotection during CABG with CPB (cardiopulmonary bypass). Patients in the IHHT group had a lower degree of serum lactate accumulation compared to the other two groups. Due to troponin dynamics, it is indicated that these patients had less myocardial damage in the postoperative period [13].

In 2019, Serebrovska et al. analyzed the effects of intermittent hypoxia–hyperoxia in the elderly with mild cognitive impairment (MCI). The 21 participants were between 51 and 74 years old and were randomly assigned into three groups as follows: seven healthy subjects were assigned to the healthy control group, six MCI patients were assigned to the MCI + sham group and eight MCI patients were assigned to the MCI + IHHT group. IHHT was performed five times a week for 3 weeks (15 sessions in total). A session consisted of four cycles of 5 min hypoxia (12% O_2_) and 3 min hyperoxia (33% O_2_). After IHHT, it was possible to notice the increase in total scores on the MoCA test, which represents a significant improvement in cognitive performance and also changes in the circulating biomarkers in the peripheral blood. In the MCI + sham group the analyzed parameters did not change. In conclusion, this study suggests that IHHT may be useful as a nonpharmacological therapy to improve cognitive function in pre-Alzheimer’s disease patients and to slow the progression of Alzheimer’s disease [14].

Later, in 2019, Bayer et al. published another study that highlighted the effectiveness of intermittent hypoxia–hyperoxia together with MTI (multimodal training intervention) on mobility and perceived health in geriatric patients. A total of 41 patients initially participated in this randomized controlled double-blind trial. Later, 34 patients remained in the study and were randomly assigned to the hypoxic group or the normoxic group. MTI included 15–20 days of therapy, being an individual plan in functions of the needs of each patient. Patients followed the treatment 2–3 times a week for 5–7 weeks. Patients in the hypoxic group received hypoxic gas mixtures with 10–14% O_2_ for 4–7 min, then hyperoxic 30–40% O_2_ for 2–4 min. Patients in the normoxic group breathed normoxic air. Thus, patients underwent a total of 12–15 hypoxic or normoxic treatment procedures, always with MTI on the same day. The treatment was well tolerated by the patients, as there were no adverse side effects, and only in rare cases sleepiness and light dizziness were reported during the hypoxic treatment. After the study, no significant difference could be revealed between the two groups. IHHT added to MTI did not produce additional improvements compared to MTI alone on health and mobility in these patients [15].

In 2021, Bestavashvili et al. published a study on the effects of intermittent hyperoxia–hypoxia on lipid profile and inflammation in patients with metabolic syndrome. The study was a prospective randomized controlled single-blind trial and followed the evolution of 65 patients with metabolic syndrome aged between 29–74 years. The 65 patients were assigned to either the IHHT group or the control group. The IHHT group consisted of 32 patients, and the control group consisted of 33 patients. Patients in the IHHT group received hypoxic (12–11% O_2_) gas mixtures for 4–7 min and hyperoxic (30–35%) gas mixtures for 2–4 min. Patients in the control group received a similar treatment, but with normoxic gas mixture (room air). The patients performed a total of 15 treatment sessions five times a week, with 2 days off on the weekend, for 3 weeks. According to this study, patients with metabolic syndrome could benefit from IHHT without any risks. The reduction in systemic inflammation and the improvement of the lipid profile reveals the effectiveness of the therapy, which is noted by the decrease in inflammatory markers and lipid metabolism in the IHHT group [16].

In a new study conducted in 2022, Chen et al. hypothesized that hypoxia–hyperoxia preconditioning in male athletes could improve muscle recovery after heavy resistance exercises by attenuating the levels of muscle damage markers.

Eleven male swimmers participated in the study. They were randomly assigned to preconditioning trials: normoxia or hypoxia–hyperoxia. Subjects in the normoxic group received FiO_2_ = 0.21, and those in the hypoxic–hyperoxic group received hypoxic (FiO_2_ = 0.10) and hyperoxic (FiO_2_ = 0.99) treatment for 60 min. After 30 min, patients performed heavy resistance exercises to induce muscle damage. After the first attempt, subjects from both groups switched and performed the same procedure. The study found that muscle injuries and pain after exercise can be alleviated with pre-exercise hypoxia–hyperoxia therapy, but no improvement was found in terms of muscle strength recovery in male swimmers [17].

In a study also conducted in 2022, physical and cognitive performance in geriatric patients was studied by Behrendt et al. They followed the effects of IHH therapy before an aerobic cycling exercise. 

Participants were randomly assigned to two groups: an intervention group (those who would receive hypoxia–hyperoxia) and a sham control group. Each session that the patients performed consisted of two parts and lasted approximately 60 min. In the first part, patients in the intervention group breathed hypoxic–hyperoxic air (FiO_2_ = 0.10–0.14 for 1–5 min interspersed with FiO_2_ = 0.30–0.40 for 1–3 min). This hypoxia–hyperoxia cycle was repeated four–eight times during a 30 min session. Patients in the control group breathed normoxic air (fraction of inspired oxygen FiO_2_ = 0.21) for 30 min. The second part consisted of aerobic cycling performed on a cycle ergometer for 20 min. Both groups trained for 6 weeks, three times a week, for a total of 18 sessions. This study suggests that hypoxia–hyperoxia before aerobic cycling exercise seems to be more effective in comparison to aerobic exercise alone regarding the increase in global cognitive functions and physical performance, but also to preserve functional mobility in geriatric patients after a 6-week intervention period [18]. 

Evidence-based literature data focused on the benefits of hypoxia–normoxia therapy (Table 3).

Burtscher et al. published a randomized double-blind study in 2004 involving physically active men with NYHA (New York Heart Association) class I and II disease, with or without prior myocardial infarction. A total of 16 patients who met the inclusion criteria (8 patients with prior myocardial infarction and 8 patients without) participated in the study, being randomly assigned to the hypoxia group or the control group. The study consisted of a 3-week breathing program, with five sessions per week. Patients in the hypoxia group underwent sessions that consisted of three to five hypoxic periods (14–10% FiO_2_), each lasting 3–5 min, and normoxic intervals lasting 3 min. Patients in the control group inhaled only normoxic air. Exposure to passive short-term intermittent hypoxia for 3 weeks increased aerobic capacity and exercise tolerance in elderly men with or without coronary artery disease [19].

In 2009, Haider et al. published an article which evaluated the effects of hypoxic training on cardiovascular and respiratory control in patients with mild COPD. This double-blind placebo-controlled study included 18 patients with mild COPD who had the following symptoms: chronic cough, sputum production, wheezing and dyspnea. The participants were randomly assigned to the training group or the placebo group, with nine people in each group. Baseline data obtained from 14 age-matched healthy subjects were used as healthy controls. Patients in the training group performed 3 weeks of IHT (interval hypoxic training) and those in the placebo group performed 3 weeks of sham training. IHT was performed daily, five times a week, for a total of 15 sessions. Each session consisted of three to five hypoxic periods (15–12% O_2_), each lasting 3–5 min, with normoxic intervals lasting 3 min. The placebo group performed a similar program but inhaled normoxic air. The conclusion of the study was that patients with mild COPD had cardiovascular autonomic abnormalities at baseline that normalized with hypoxic training. The absence of adverse side reactions and the autonomic improvement suggest that IHT may be a therapeutic strategy in COPD, but it requires studies in more compromised patients as well [20].

Burtscher et al., also in 2009, published a study in which they analyzed the effects of repeated short-term hypoxia on exercise tolerance in patients at risk or with mild COPD. They included in their double-blind study 18 patients, 10 were men and 8 women. Patients were active people with COPD symptoms and were randomly assigned to two groups: the hypoxia group or the control group. Patients underwent the 3-week breathing program consisting of five sessions per week. Sessions consisted of three to five hypoxic periods lasting 3–5 min and normoxic periods lasting 3 min. Patients in the control group performed the program by inhaling normoxic air. Intermittent hypoxia appears to improve exercise tolerance by increasing total hemoglobin mass and diffusing capacity for carbon monoxide (DLCO) in the studied patients [21].

In 2012, Mekjavic et al. published a study that investigated the effect of daily resting intermittent hypoxic exposures (IHE) on peak aerobic capacity and performance under normoxic and hypoxic conditions. Eighteen young, healthy males participated in the study and were equally assigned to the control group or the IHE group. The subjects in both groups performed endurance exercise training on a cycle ergometer. The subjects in the IHE group performed the IHE protocol prior to each exercise training session. The protocol was performed five times a week for 4 weeks for a total of 20 sessions. Each session comprised a period (4 min) during which subjects breathed room air, followed by seven IHE cycles. A cycle consisted of 5 min of hypoxia (fraction of inspired oxygen FiO_2_ = 0.12–0.9) and 3 min of normoxia (FiO_2_ = 0.21). The study concluded that the IHE protocol did not improve performance or peak aerobic capacity in hypoxia or normoxia during and immediately after the protocol [22].

In 2014 Kon et al. published a randomized trial involving 16 healthy, non-smoking male subjects. They analyzed the effects of resistance exercise training under systemic hypoxia on the angiogenic response and muscle endurance in human skeletal muscle. The subjects were assigned to either the normoxic resistance training group (NRT group—7 people) or the hypoxic resistance training group (HRT group—9 people). Participants performed 8-week resistance training (on non-consecutive days), 16 sessions in total. Except for the 1st and the 16th sessions, subjects in the HRT group were exposed to normobaric hypoxia (14.4% O_2_) from 10 min before the resistance exercise session until 30 min after the resistance exercise session. During the 1st and 16th sessions the subjects were exposed to hypoxia from 15 min before the resistance exercise session until 60 min after the resistance exercise session. The study demonstrated greater increases in muscular endurance and angiogenesis in skeletal muscle following resistance training under systemic hypoxia [23].

Lizamore et al. published a study in 2016 that assessed the instantaneous and adaptive effects of 4 weeks of IHE on resting heart rate variability (HRV) in a sedentary population. The study included 16 participants who were randomly assigned to the group receiving IHE—Hyp group—or to the placebo group—C group—with 8 people in each group. Participants remained blind to their groups throughout the study. For 4 weeks, four times per week during seated rest, the study participants received six 5 min intervals of hypoxic or placebo air interspersed with six 5 min periods of normoxic ambient air. Hypoxic air was controlled as the following for the Hyp group: week 1, partial saturation of oxygen SpO_2_ = 95% (fraction of inspired oxygen FiO_2_ ~ 0.21); week 2, 90% (FiO_2_ ~ 0.16); week 3, 85% (FiO_2_ ~ 0.13) and week 4, 80–85% (FiO_2_ ~ 0.10–0.12). Group C patients had SpO_2_ > 95% throughout the study. The authors concluded that four weeks of IHE is unlikely to improve maximal exercise capacity, but it may be useful in increasing HRV in people who cannot exercise [24].

In 2020, Bao et al. published a single-blind randomized controlled trial in which they studied the effects of intermittent hypoxia training (IHT) for migraine. The 48 patients were randomly divided as follows: 24 participants in the IHT group and 24 in the control group. Patients in the IHT group were exposed to five cycles of 10% O_2_ for 5 min, followed by 5 min of room air. Each session lasted 50 min and five sessions were performed per week for 8 weeks. There were significant differences between the two groups on the parameters analyzed in the end of 8 weeks. Attack frequency was improved within 3 months in patients in the IHT group. IHT could be an effective method for migraine patients, as it could improve migraines up to 3 months after the intervention [3]. 

**Figure 2:**
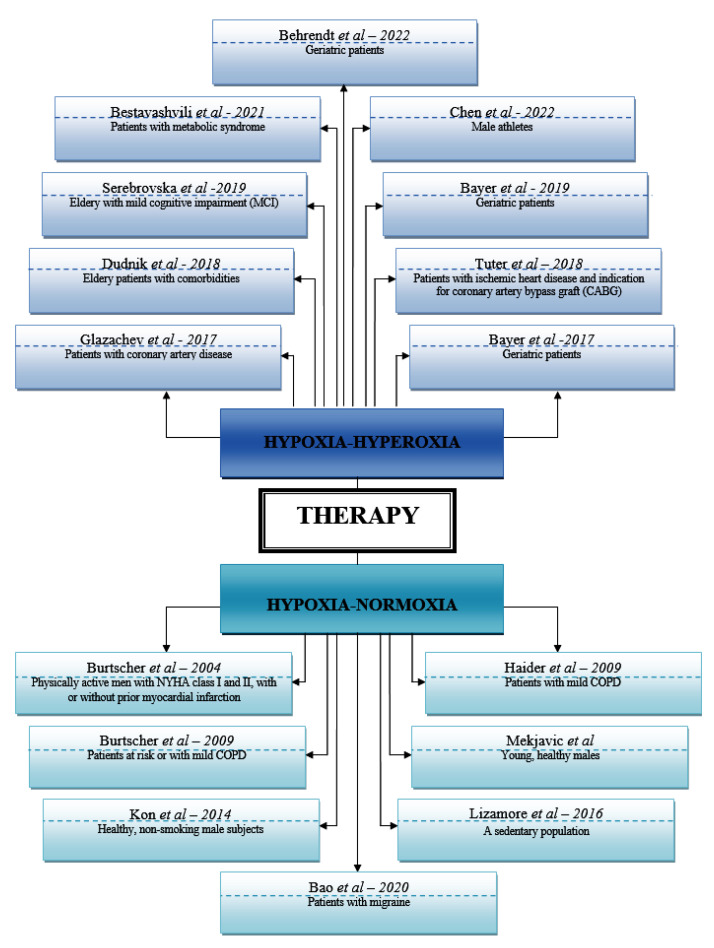
The articles, the authors, year of publication and the pathologies studied [3, 9,10,11,12,13,14,15,16,17,19,20,21,22,23,24].

In Figure 2 we illustrate the articles in which hypoxia–hyperoxia and hypoxia–normoxia therapies were used, the authors of the studies, the year of publication and the pathologies that were studied.

## 4. Discussion

Intermittent hypoxia–hyperoxia training (IHHT) can bring important benefits in improving the symptoms and functionality of patients with various cardiovascular, respiratory, musculoskeletal, neurological, and metabolic pathologies and also geriatric patients with multiple comorbidities. These patients may have contraindications to multimodal therapy, which also includes physiotherapy, considering that many of them have multiple organ failures (kidney, liver, and heart failure) [25,26].

Published studies demonstrate that IHHT brings superior improvements to multimodal therapy (physiotherapy, physical therapy, and occupational therapy), demonstrating its effects at the biological and molecular level, but also at the functional level, of the cardiorespiratory fitness and cognitive status of the patients [5,27].

Specific systemic mechanisms of adaptation to hypoxia include changes in the functioning of the cardiovascular system that increase oxygen delivery to tissues in need, changes in pulmonary ventilation and changes at the tissue level that allow more efficient use of oxygen for metabolic processes. Specific reactions to hypoxia are accompanied by an increase in the blood level of glucocorticoids. This adaptive response provides increased resistance to hypoxia and many other environmental factors [28].

Combining hypoxia and hyperoxic breaks in one procedure has a good physiological basis in the hypoxic–hyperoxic paradox hypothesis. Hypoxia is a natural trigger of mitogenesis and mitochondrial metabolic changes by inducing hypoxia-inducible factor (HIF), vascular endothelial growth factor (VEGF), other relevant molecular cascades, stem cell proliferation, etc. Hyperoxic stimuli accompanied by increased oxygen availability promote the production of both reactive oxygen species (ROS) and ROS scavengers and trigger the same molecular cascades as hypoxia, activating angiogenesis, mitogenesis, oxidative phosphorylation (OXPHOS) efficiency and metabolic activity in various tissues [16].

Acute hypoxia causes mitochondrial swelling, organelle vacuolization and disorganization and destruction of mitochondrial membranes. Exposure to IHT causes an increase in the total number of mitochondria, a reduction in the number of structurally modified organelles, the appearance of energetically active Mt with vesicular cristae and the formation of micromitochondria (microMt). Excessive production of reactive oxygen species (ROS) in the mitochondria that oxidize proteins, lipids and DNA is an important mechanism of cell damage during hypoxia and reoxygenation. The low level of ROS production is protective and serves as a trigger for adaptive responses. IHT leads to the reprogramming of mitochondrial metabolism, ensuring adequate ATP production. Activation of potassium transport in the mitochondrial matrix under IHT is a protective mechanism against Ca^2+^ overload caused by acute hypoxia. All hypoxia adaptation reactions are regulated by HIF factors (HIF-1, HIF-2 and HIF-3). Each of the HIF subunits plays a specific role depending on the mode of hypoxia-induced stress [29].

The 2019 Nobel Prize in Physiology or Medicine was awarded to three physician scientists, Drs. William G. Kaelin, Jr., Peter Ratcliffe and Gregg Semenza, for their groundbreaking work revealing how cells sense and adapt to oxygen availability [30].

In 1995, Dr. Guang-Liang Wang and Dr. Bing-Hua Jiang purified and cloned this transcription factor, which they named hypoxia-inducible factor-1 (HIF-1). HIF-1 is composed of two subunits, HIF-1α and HIF-1β. Despite the fact that mRNA expression of both subunits typically remains constant in either normoxia or hypoxia, the HIF-1α protein was found to be induced and accumulate in hypoxia, suggesting that some type of post-transcriptional/post-translational modifications contribute to the regulation of HIF-1α at the protein level [30].

Biological responses to intermittent hypoxia can be either adaptive or maladaptive, depending on the severity, frequency, and duration of hypoxemia. The different detrimental versus beneficial effects of intermittent hypoxia may depend on the different number of hypoxia episodes, severity and total exposure duration of hypoxia, which may mobilize the body’s adaptive mechanisms or cause dangerous pathological processes in more severe or prolonged hypoxia events. Potential beneficial effects of IHT on the human cardiovascular system have been either experimentally demonstrated or proposed, including the following: increased myocardial metabolic processes, increased myocardial tolerance to ischemia-reperfusion injury, reduction in free radical damage at the cellular level, development of endothelial function and microcirculation, positive inotropic effect on cardiac function, normalization of blood pressure, reduction in sympathetic nervous system activity, limitation of blood viscosity and platelet aggregation [31].

Low doses of IH upregulate hypoxia-sensitive growth/trophic factors within respiratory motoneurons but do not cause detectable pathologies such as hippocampal cell death, neuroinflammation or systemic hypertension. Progress has been made in understanding the cellular mechanisms that give rise to IH-induced respiratory plasticity, and attempts have been made to harness the benefits of low-dose IH to treat respiratory failure after cervical spine injury [32].

In 2014, Wang et al. designed a study in which they followed weight loss in obese adolescents by exposure to IH for 4 weeks together with physical training and dietary intervention. They intended to include in the study 40 obese girls and boys aged between 11–15 years. They were to be assigned to the control group (sleep under normal conditions) or the hypoxia group (sleep in a normobaric hypoxia room). The results could lead to a potential utility of IHT in a weight loss program among obese children and adolescents [33].

Humans or rodents exposed short-term to moderate isocapnic hypoxemia (SaO_2_ 75–80%) daily sessions for several weeks, with 10–15 episodes lasting 1–2 min alternating with an equal duration of normoxia, may benefit without maladaptive cardiovascular sequelae. The improvement of ventilation in rodents and humans with spinal cord injuries can be achieved with this type of noncyclic, short-duration, mild-severity IH, which induces respiratory motoneuron plasticity, followed by a progressive increase in phrenic and hypoglossal nerve [34]. 

Another important concept of adaptation to intermittent hypoxia is its impact on extracellular adenosine generation and signaling. Adenosine belongs to the group of molecules called purines. Purines are some of the most influential and oldest biochemical compounds in the history of evolution. Alan Drury and Albert Szent-Györgyi of the University of Cambridge suggested in 1929 that purines might also function as extracellular signaling molecules. In many studies, it is suggested that extracellular adenosine comes mainly from the breakdown of precursor nucleotides (for example ATP—adenosine triphosphate) under certain conditions such as hypoxia, inflammation or ischemia. The hypoxia–adenosinergic signaling pathway can be targeted to make cancer-adoptive immunotherapy, according to Sitkovsky et al. Dr. Colgan’s studies implicated inflammatory hypoxia in the extracellular production and control of adenosine signaling and identified hypoxia-induced increases in adenosine signaling as a control mechanism for attenuating intestinal inflammation, as occurs during inflammatory bowel disease [35].

To understand the interactions between PMNs (polymorphonuclear leukocytes) and the vascular endothelium and to identify potential therapeutic targets to limit the vascular leakage syndrome associated with inflammation and hypoxia, the first step is to identify the biological mechanisms that control endothelial barrier function and regulate vascular leakage. Hypoxia is a common feature of inflamed tissues, accompanied by significantly increased levels of adenosine. The exact source of adenosine is not well defined, but it may result from a combination of increased intracellular metabolism and the enhanced extracellular phosphohydrolyzing of adenine nucleotides by surface ecto-nucleotidases [36].

Hypoxia-inducible factors (HIFs) are found in inflammatory conditions and diseases, including inflammatory bowel disease, pathogen infection, acute lung injury and myocardial injury, or during ischemia-reperfusion injury. Hypoxia and inflammation are closely related: at the cellular level, hypoxia can cause inflammation, and inflammation can cause hypoxia. Although hypoxia can be an inflammatory stimulus that encourages pro-inflammatory responses and destroys tissue barriers, there are many examples where stabilization of HIFs induces anti-inflammatory and tissue-protective responses [37].

MicroRNAs (miRNAs—micro ribonucleic acid) are considered to be functionally involved in almost all physiological processes, including differentiation and proliferation, hemostasis, metabolism, apoptosis and inflammation. Transcriptional regulation of miRNA expression can be controlled by classical transcription factors. In hypoxia, the hypoxia-inducible transcription factor (HIF) is stabilized and has been shown to regulate a group of miRNAs. The interactions between miRNA and HIF are feedback loops that are relevant to cellular processes such as proliferation, cell cycle progression or angiogenesis, processes that play a role in tumorigenesis but also in ischemia-reperfusion [38].

Despite the wide range of possible effects on various pathological conditions (Table 4), IH is not routinely prescribed or widely used. Future studies on larger populations on the efficacy and long-term effects of IH are needed in order to gain more evidence for further recommendations.

## 5. Conclusions

This review highlights the merits of hypoxic–hyperoxic and hypoxic–normoxic therapy in various conditions and populations. The existing literature indicates that protocols consisting of exposure to a mild level of hypoxia for a small number of short episodes can lead to significant beneficial results if administered over several days or weeks. These therapies can be a non-pharmacological treatment alternative if the risk of intermittent hypoxia protocols is correctly assessed. Although these results appear to be promising, further research focused on the benefit and optimal dose of IHHT and IHT are awaited, as the literature is currently heterogenous and insufficient.

This therapy proved to be safe and effective in the patients included in the studies presented above. Hypoxia–hyperoxia and hypoxia–normoxia seem to be promising non-pharmacological intervention strategies well tolerated by patients. These therapies have benefited geriatric patients, patients with coronary artery disease, patients with mild cognitive impairment, with metabolic syndrome, geriatrics with comorbidities, male athletes, patients with ischemic heart disease and indication for CABG, physically active men with NYHA class I/II with or without prior myocardial infarction, COPD patients, sedentary populations, healthy and non-smoking subjects, migraine patients and healthy male subjects. Therefore, it can be assumed that these therapies can be included in the therapeutic management of various pathologies.

## Figures and Tables

**Table 1:** The keyword combinations used for the contextual searches in the international databases.

	PubMed	Elsevier	Cochrane	PEDro	Total
Intermittent hypoxia–hyperoxia	29	85	33	3	150
Intermittent hypoxia–hyperoxia training	5	110	21	3	139
Intermittent hypoxic training	34	487	107	6	634
Total	68	682	161	12	923

**Table 2:** Centralized clinical studies performed on the effect of intermittent hypoxia–hyperoxia on various pathologies.

Authors	Country	Year	Study Design	No. pac incl	References
Glazachev et al.	Russia	2017	non-randomized controlled before-and-after trial	46	21
Bayer et al.	Austria	2017	double-blind, randomized, stratified and placebo-controlled study	34	46
Dudnik et al.	Russia	2018	randomized controlled trial	28	16
Tuter et al.	Russia	2018	single-center, randomized controlled trial	120	29
Serebrovska et al.	Ukraine	2019	a pilot study	21	73
Bayer et al.	Austria	2019	double-blind, randomized controlled clinical trial	34	46
Bestavashvili et al.	Russia	2021	single-center, single-blind, randomized controlled trial	65	36
Chen et al.	Taiwan	2022	randomized, double-blind study	11	53
Behrendt et al.	Germany	2022	randomized, two-armed, controlled and single-blinded trial	28	164

Abbrev: No. pac incl—number of patients included.

**Table 3:** Centralized clinical studies performed on the effect of hypoxia–normoxia on various pathologies.

Authors	Country	Year	Study Design	No. pac incl	References
Burtscher et al.	Austria	2004	randomized, double-blind	16	37
Haider et al.	Austria	2009	randomized, double-blind, placebo-controlled study	18	43
Burtscher et al.	Austria	2009	randomized, double-blind	18	42
Mekjavic et al.	Slovenia	2012	not mentioned	18	36
Kon et al.	Japan	2014	a pilot study	16	34
Lizamore et al.	New Zealand	2016	Single-blind study	16	30
Bao et al.	China	2020	prospective, assessor-blinded randomized controlled trial	48	30

**Table 4:** Effects of intermittent hypoxia—IH—on different clinical conditions [35].

Pathology	Effect Observed
Chronic obstructive pulmonary disease	In randomized, double-blind, controlled clinical studies it was demonstrated that mild repetitive acute IH (12–15% O2 for 3–5 min, followed by intervals of 3–5 min of normoxia, 5–9 episodes per day, for 15 days) can produce the following increases: exercise time, baroreflex sensitivity, total hemoglobin, hypercapnic ventilatory response, forced vital capacity and forced expiratory volume in 1 s.
Arterial hypertension	In 56 known patients with stage I-II hypertension, moderate IH reduced heart rate, systolic and diastolic blood pressure and peripheral resistance. IH reduces the symptoms of angina, normalizes microcirculation and lipid metabolism and increases maximal oxygen consumption and exercise tolerance, being proven to be a safe therapy for elderly patients. Increased endothelial NO production that produces the opening of reserve capillaries and vasodilatation can determine the antihypertensive effects of moderate IH (reduced peripheral resistance), reduced sympathetic activity, minimized calcium overload of vascular smooth muscles, improved water and salt metabolism, increased activity of antioxidant enzymes and increased synthesis of angiogenic growth factors, including VEGF and FGF.
Myocardial Infarction	In humans, moderate IH increases maximal oxygen consumption in older men (50–70 years), both with and without coronary artery disease. During submaximal exercise (cycling at 1 W/kg), systolic blood pressure, heart rate, perceived exertion and blood lactate concentration are diminished by IH. Myocardial protection is correlated with the ability of moderate IH to increase coronary blood flow, myocardial vascularity, cardiomyoglobin and antioxidant enzyme expression. IH increases erythropoietin (EPO) concentrations, stimulating erythropoiesis and increasing hematocrit, blood viscosity and platelet count.
Inflammatory/immune responses to IH	Some studies suggest that moderate IH protocols enhance the innate immune system while having a general anti-inflammatory effect. For example, in healthy humans, exposure to the 4 5 min episodes of 10% O_2_ (5 min interval in room air, 14 days) increases the phagocytic and bactericidal activities of neutrophils, while suppressing the pro-inflammatory mediators TNF-α and IL-4 by more than 90%. These responses, which persisted at least 7 days after IH, may increase the body’s immune defenses without associated inflammation.
Metabolic responses to IH	IH protocols have beneficial effects on metabolism, including decreased body weight, cholesterol, and blood sugar levels, as well as increased insulin sensitivity. Mechanisms of moderate IH-induced weight loss may include increased serotonin and/or leptin levels. Body weight is reduced with moderate IH by increasing hepatic leptin expression and increasing blood leptin concentration. Moderate hypoxia (14.6% O_2_) reduces cholesterol and blood glucose and increases insulin sensitivity in patients with type 2 diabetes. Hypoxia also increases glycolysis and fatty acid oxidation and mitochondrial enzyme activity and reduces cholesterol synthesis.
Bone	IH has positive effects on bone tissue remodeling. Exposure of rats to IH determined high alkaline phosphatase activity in bone tissue, thus suggesting increased osteoblast activity and new bone formation.

## Data Availability

Not applicable.
